# Trends in seroprevalence of SARS‐CoV‐2 and infection fatality rate in the Norwegian population through the first year of the COVID‐19 pandemic

**DOI:** 10.1111/irv.12932

**Published:** 2021-11-09

**Authors:** Gro Tunheim, Gunnar Øyvind Isaksson Rø, Trung Tran, Anne‐Marte Bakken Kran, Jan Terje Andersen, Eline Benno Vaage, Anette Kolderup, John Torgils Vaage, Fridtjof Lund‐Johansen, Olav Hungnes

**Affiliations:** ^1^ Division of Infection Control Norwegian Institute of Public Health Oslo Norway; ^2^ Department of Immunology Oslo University Hospital Rikshospitalet, University of Oslo Oslo Norway; ^3^ Department of Pharmacology, Institute of Clinical Medicine University of Oslo Oslo Norway; ^4^ ImmunoLingo Convergence Centre University of Oslo Oslo Norway

**Keywords:** COVID‐19, infection fatality rate, infection hospitalization rate, Norway, SARS‐CoV‐2, seroprevalence

## Abstract

**Background:**

Infection with the novel coronavirus SARS‐CoV‐2 induces antibodies that can be used as a proxy for COVID‐19. We present a repeated nationwide cross‐sectional study assessing the seroprevalence of SARS‐CoV‐2, the infection fatality rate (IFR), and infection hospitalization rate (IHR) during the first year of the pandemic in Norway.

**Methods:**

Residual serum samples were solicited in April/May 2020 (Round 1), in July/August 2020 (Round 2) and in January 2021 (Round 3). Antibodies against SARS‐CoV‐2 were measured using a flow cytometer‐based assay. Aggregate data on confirmed cases, COVID‐19‐associated deaths and hospitalizations were obtained from the Emergency preparedness registry for COVID‐19 (Beredt C19), and the seroprevalence estimates were used to estimate IFR and IHR.

**Results:**

Antibodies against SARS‐CoV‐2 were measured in 4840 samples. The estimated seroprevalence increased from 0.8% (95% credible interval [CrI] 0.4%–1.3%) after the first wave of the pandemic (Rounds 1 and 2 combined) to 3.2% (95% CrI 2.3%–4.2%) (Round 3). The IFR and IHR were higher in the first wave than in the second wave and increased with age. The IFR was 0.2% (95% CrI 0.1%–0.3%), and IHR was 0.9% (95% CrI 0.6%–1.5%) for the second wave.

**Conclusions:**

The seroprevalence estimates show a cumulative increase of SARS‐CoV‐2 infections over time in the Norwegian population and suggest some under‐recording of confirmed cases. The IFR and IHR were low, corresponding to the relatively low number of COVID‐19‐associated deaths and hospitalizations in Norway. Most of the Norwegian population was still susceptible to SARS‐CoV‐2 infection after the first year of the pandemic.

## 1 INTRODUCTION

**FIGURE 1:**
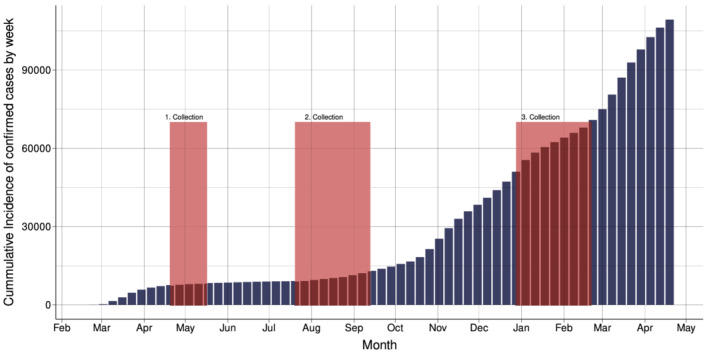
Cumulative incidence of polymerase chain reaction (PCR)‐confirmed COVID‐19‐cases in Norway in 2020 and 2021 by month as reported to the Norwegian Surveillance System for Communicable Diseases (MSIS). The first collection (Round 1) of residual serum samples occurred in April/May 2020 (week numbers 17–20), the second collection (Round 2) between July and September 2020 (week numbers 30–37) and the third collection (round 3) between December 2020 and February 2021 (week numbers 53–6)

On 11 March 2020, the World Health Organization (WHO) assessed that coronavirus disease 2019 (COVID‐19) should be characterized as a pandemic.^1^ COVID‐19 is caused by severe acute respiratory syndrome coronavirus 2 (SARS‐CoV‐2), a novel virus belonging to the coronavirus family. Norway, with a population of 5.4 million and 1409/100,000 cases and 11.8/100,000 deaths confirmed by 7 March 2021, has not been as severely affected as many other European countries.^2^ In Norway, the first COVID‐19 case was confirmed on February 26th, 2020.^3^ Subsequently, there was a rise in confirmed cases with a peak incidence in March 2020 (Figure 1). On 12 March, the Norwegian government issued a lockdown,^4^ and the incidence of cases diminished.^2^ From May 2020, there was a gradual re‐opening of the Norwegian society, but the pandemic was quite contained during the summer with few new confirmed cases. In the autumn of 2020, the numbers of confirmed COVID‐19 cases again started rising, representing a second wave of the pandemic, a pattern also observed in other European countries.^2^, ^5^ In the first week of January 2021, there was a new peak in the incidence of COVID‐19 cases in Norway, followed by a decline probably due to additional non‐pharmaceutical measures. On 27 December 2020, the first dose of COVID‐19 vaccine was administered in Norway.^2^

Infection with SARS‐CoV‐2 induces antibodies to the virus; therefore, the presence of these antibodies in a person's blood, in the absence of vaccination, can be used as a proxy for SARS‐CoV‐2 infection. Studies have shown that most individuals seroconvert within 2 weeks of infection.^6^

We here present a repeated, nationwide cross‐sectional study with three sampling rounds that shows the changes in the seroprevalence of SARS‐CoV‐2 in Norway during the first year of the COVID‐19 pandemic. In addition, we investigated the potential background seroprevalence against SARS‐CoV‐2 in pre‐pandemic sera from 2019. We have also used the seroprevalence estimates to assess the infection fatality rate (IFR) and infection hospitalization rate (IHR) after the first and second wave of the pandemic in Norway.

## 2 METHODS

### 2.1 Study samples

Panels of anonymized residual serum samples were solicited across Norway according to a scheme for annual serosurveillance of influenza in Norway.^7^ In order to study the exposure to SARS‐CoV‐2 at a population level, we collected 900 sera from 9 microbiological laboratories in the spring of 2020 (Round 1), 1812 sera from 16 laboratories in the late summer of 2020 (Round 2) and 1912 sera from 17 laboratories in January 2021 (Round 3) (Table 1). In addition, 216 pre‐pandemic residual sera collected by the annual scheme in August 2019 were analysed to study potential cross‐reacting antibodies in Norwegian residual sera. In the first sampling round, the laboratories were asked to avoid samples from individuals with suspected COVID‐19, or samples submitted for COVID‐19 analysis, but this was not the case for Rounds 2 and 3.

**TABLE 1:** Overview of the number of residual serum samples, the number of samples positive for antibodies against SARS‐CoV‐2 and estimated seroprevalence for all three sampling rounds

	Round 1, April/May 2020 (week no. 17‐20^a^)			Round 2, late summer 2020 (Round 2, week no. 30‐37^b^)			Round 3, January 2021 (week no. 53, 2020–6, 2021^c^)		
	Number of samples collected (%)	Positive samples (%)	Estimated seroprevalence, % (95% CrI)	Number of samples collected (%)	Positive samples (%)	Estimated seroprevalence, % (95% CrI)	Number of samples collected (%)	Positive samples (%)	Estimated seroprevalence, % (95% CrI)
Overall^d^	900 (100)	10 (1.1)	1.0 (0.1–2.4)	1812 (100)	11 (0.6)	0.6 (0.2–1.2)	1912 (100)	61 (3.2)	3.2 (2.3–4.2)
**Sex**									
Male	383 (42.6)	6 (1.6)	1.6 (0.3–3.6)	770 (42.5)	6 (0.8)	0.9 (0.3–1.9)	827 (43.3)	36 (4.4)	4.4 (3.0–6.1)
Female	509 (56.6)	4 (0.8)	0.8 (0.1–2.1)	1039 (57.3)	5 (0.5)	0.6 (0.2–1.3)	1077 (56.3)	25 (2.3)	2.3 (1.4–3.4)
Missing	8 (0.9)	0	n.a^e^	3 (0.2)	0	n.a.	8 (0.4)	0	n.a.
Age groups									
0–4 years	41 (4.6)	0	1.9 (0.1–9.6)	112 (6.2)	0	0.7 (0.0–3.8)	158 (8.3)	9 (5.7)	6.3 (2.8–10.7)
5–14 years	115 (12.8)	2 (1.7)	2.3 (0.2–6.8)	312 (17.2)	3 (1.0)	1.3 (0.3–3.2)	335 (17.5)	14 (4.2)	4.4 (2.3–6.9)
15–24 years	166 (18.4)	2 (1.2)	1.5 (0.2–4.7)	308 (17.0)	3 (1.0)	1.3 (0.3–3.2)	320 (16.7)	8 (2.5)	2.7 (1.1–4.9)
25–59 years	372 (41.3)	4 (1.1)	1.1 (0.1–2.9)	700 (38.6)	2 (0.3)	0.4 (0.0–1.2	708 (37.0)	15 (2.1)	2.1 (1.1–3.4)
≥60 years	206 (22.9)	2 (1.0)	1.2 (0.1–3.8)	379 (20.9)	3 (0.8)	1.0 (0.2–2.6)	391 (20.4)	15 (3.8)	4.0 (2.2–6.2)
Missing	—	—	—	1 (0.1)	0	n.a.	—	—	—

^a^ 55.6% of samples were collected in week no. 19, 2020.

^b^ 45.3% of samples were collected in week no. 31, 2020.

^c^ 55.6% of samples were collected in week no. 1, 2021.

^d^ The overall seroprevalence for Norway was adjusted for age, sex and county.

^e^ n.a.: not applicable. The seroprevalence was not estimated for subgroups with less than 30 samples tested.

### 2.2 Antibody analysis

The residual sera were analysed using an in‐house flow cytometer‐based method detecting IgG antibodies against SARS‐CoV‐2‐derived recombinant antigens.^8^ Samples with antibodies against both the receptor‐binding domain (RBD) and the nucleocapsid protein of SARS‐CoV‐2 were considered positive.

### 2.3 Sensitivity and specificity

Because the analysis was under active development between the collection rounds, we present seroprevalence estimates for each round with estimates of sensitivity and specificity at the time of data collection. This gives a sensitivity of 86% (95% credible interval [CrI] 74%–94%) and specificity of 100% (95% CrI 99%–100%) for Round 1, a sensitivity of 86% (CrI 82%–90%) and specificity of 99.9% (95% CrI 99.7–100.0%) for Round 2 and a sensitivity of 96% (95% CrI 94%–98%) and specificity of 99.8% (95% CrI 99.5%–99.9%) for Round 3. Sensitivities were estimated, based on the detection of antibodies against SARS‐CoV‐2 in serum samples collected at least 3 weeks from onset of symptoms from individuals with polymerase chain reaction (PCR)‐confirmed COVID‐19. For the first two rounds, specificity was estimated from the proportion of negative samples among the true negative pre‐pandemic samples, and for Round 3, the specificity was estimated in a validation panel by reanalysing all test‐positive sera with the Roche Elecsys® Anti‐SARS‐CoV‐2 antibody test. Test sensitivity and specificity have been used to convert proportions of test positives into estimated seroprevalences.

### 2.4 Statistical methods

Seroprevalence was estimated overall for Norway and by sex, age group and county of residence (11 in total). For the estimation, we used a Bayesian method that incorporates the uncertainties in the sensitivity and the specificity of the test.^9^ We adjusted the overall seroprevalence by a multilevel regression and poststratification on counties, age groups, and sex.^9^ For seroprevalence results, we present a point estimate and a 95% CrI. The seroprevalence was not estimated for subgroups with less than 30 samples tested. Seroprevalence data on county of residence are detailed elsewhere.^10^, ^11^, ^12^

A seroprevalence estimate for ‘Wave 1’ (July 2020) was obtained by combining the data from Rounds 1 and 2 as there was a short time between these two collections and there were few new confirmed cases, COVID 19‐associated deaths or hospital admissions between these collections. The combined seroprevalence was estimated as above by pooling of all samples from the first two rounds. All Bayesian analyses were performed using Stan^13^ with the RStan interface.^14^

### 2.5 IFR and IHR

Aggregate data on COVID‐19‐associated deaths and hospitalizations were obtained from the Emergency preparedness register for COVID‐19 (Beredt C19), which includes linked data from the Norwegian Surveillance System for Communicable Diseases (MSIS) and the Norwegian Intensive Care and Pandemic Registry. COVID‐19 associated deaths are defined as deaths reported by doctors to MSIS as due to COVID‐19 or as deaths with COVID‐19 as the cause of death on the death certificate. We extracted the total number of confirmed cases, hospital admissions, and fatalities by age or sex for the first and second wave of COVID‐19 corresponding to the serum collections. We included all patients that tested positive for SARS‐CoV‐2 until 2 weeks prior to the middle of sampling Round 2 (13th of July 2020, i.e., after Wave 1) and 3 (21 December 2020, that is, after Wave 2) as reported to MSIS. Exact numbers are not provided for groups smaller than five for data privacy reasons. The estimated number of infections is then given by the estimated seroprevalence multiplied by the size of the population. We estimate the number of cases in ‘Wave 2’ (December 2020) by subtracting the combined seroprevalence after Rounds 1 and 2 from the seroprevalence after Round 3.

### 2.6 Percentage of infections detected

We calculated the percentage of infections detected from the ratio of the number of confirmed cases in MSIS to the estimated number of infections from the seroprevalence data. As for the IFR and IHR calculations, we included confirmed cases until 2 weeks prior to the middle of sampling Rounds 2 and 3.

### 2.7 Ethics

The residual sera were collected anonymously, and aggregated data on confirmed cases and COVID‐19‐associated deaths and hospitalizations were obtained from MSIS and Beredt C19, respectively. The study was approved by The Regional Committee for Medical and Health Research Ethics in South Eastern Norway (Case number 157792).

## 3 RESULTS

A total of 4624 residual sera were collected over three sampling rounds during the first year of the COVID‐19 pandemic in Norway (Table 1). The samples were tested for antibodies against SARS‐CoV‐2. In addition, 216 pre‐pandemic residual sera were included to study any potential background seropositivity.

**FIGURE 2:**
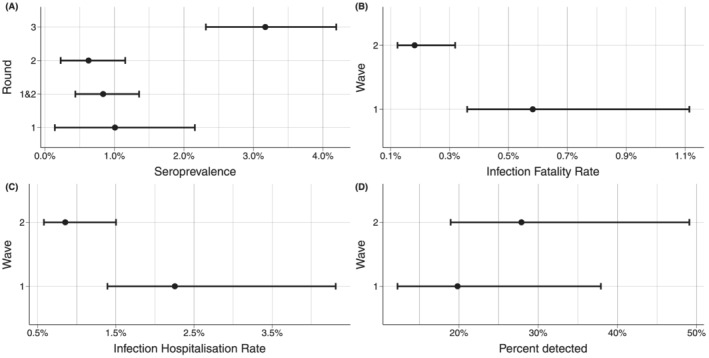
Overall seroprevalence, IFR, IHR and percentage detected infections by rounds/waves with 95% credible intervals. (A) Shows the overall estimated seroprevalence for the Norwegian population by round, (B–D) shows the overall infection fatality rate (IFR), infection hospitalization rate (IHR) and percentage of infections detected by Waves 1 and 2, respectively

None of the pre‐pandemic residual sera had antibodies cross‐reacting to SARS‐CoV‐2. In April/May 2020 (Round 1), only 10 samples were positive for antibodies against SARS‐CoV‐2 (Table 1). Based on this finding, the seroprevalence was estimated to be 1.0% (95% CrI 0.1%–2.4%) (Table 1 and Figure 2A) in the spring of 2020.^10^ There were no differences in the seroprevalence estimates between age groups or men and women (Table 1). In the late summer of 2020 (Round 2), the number of positive samples was 11 (Table 1), resulting in a slightly lower seroprevalence estimate than in the spring (0.6% [95% CrI 0.2%–1.2%]) (Table 1 and Figure 2A).^11^ Again, there were no differences in the seroprevalence estimates between age groups or men or women (Table 1 and Figure 2). According to the number of confirmed cases of COVID‐19 reported to MSIS, the pandemic was quite contained in Norway during the summer of 2020, after the peak of the pandemic in March.^2^ Therefore, we combined the data from Rounds 1 and 2 to obtain a more robust seroprevalence estimate with a larger sample size representing the seroprevalence estimate after the first wave of the pandemic in Norway. This combined seroprevalence estimate was 0.8% (95% CrI 0.4%–1.3%) (Figure 2A).

In January 2021 (Round 3), 61 samples were positive for SARS‐CoV‐2 antibodies. Thus, almost a year after the first case of COVID‐19 was reported in Norway and at the end of the second wave of the pandemic, the estimated seroprevalence had increased to 3.2% (95% CrI 2.3%–4.2%)^12^ (Table 1 and Figure 2A). As for the first two sampling rounds, there were not any clear differences between age groups in Round 3 (Table 1); however, males had higher seroprevalence than females (4.4% and 2.3%, respectively).

The estimated seroprevalence was used to calculate IFR (Figure 2B) and IHR (Figure 2C) associated with COVID‐19 for the first and the second wave of the pandemic in Norway. Corresponding to the combined Rounds 1 and 2 collection, 262 individuals had died, and 1013 individuals had been hospitalized due to COVID‐19 by July 2020 (Wave 1) (Table 2). From July to the end of December 2020 (Wave 2) another 226 individuals had died, and 1063 additional individuals had been admitted to hospital. This gives an IFR of 0.6% (95% CrI 0.4%–1.1%) during Wave 1, and 0.2% (95% CrI 0.1%–0.3%) during Wave 2. For hospitalizations, we find an IHR of 2.3% (95% CrI 1.4%–4.3%) during Wave 1, and 0.9% (95% CrI 0.6%–1.5%) during Wave 2. Figure 2D shows the percentage of infections detected for the two waves. The point estimate indicates that 19.9% (95% CrI 12.3%–38.3%) and 27.8% (95% CrI 19.0%–48.2%) were detected in Wave 1 and 2, respectively.

**TABLE 2:** COVID‐19 associated deaths and hospitalizations and confirmed cases of COVID‐19 by wave, sex and age group

	Deaths^a^ ^,^ ^b^ (Wave 1)	Deaths^a^ ^,^ ^c^ (Wave 2)	Hospitalization^a^ ^,^ ^b^ ^,^ ^d^ (Wave 1)	Hospitalization^a^ ^,^ ^b^ (Wave 2)	Confirmed cases (Wave 1)^b^ ^,^ ^d^ ^,^ ^e^	Confirmed cases (Wave 2)^c^ ^,^ ^d^ ^,^ ^e^
Total	262	226	1013	1063	8919	34 745
Sex						
Male	141	115	601	626	4420	18 534
Female	121	111	412	437	4493	16 211
Age groups						
0–4 years	<5^e^	<5	5	8	89	731
5–14 years	<5	<5	<5	9	279	3505
15–24 years	<5	<5	16	37	1024	7196
25–59 years	12	12	469	459	5488	19 304
≥60 years	250	211	522	550	2036	4012

^a^ Data from the Emergency preparedness registry for COVID‐19 (Beredt C19).

^b^ By 13 July 2020.

^c^ By 21 December 2020.

^d^ Missing data in groups.

^e^ Exact numbers are not provided for groups smaller than five for data privacy reasons.

^f^ Data from the Norwegian Surveillance System for Communicable Diseases (MSIS).

**FIGURE 3:**
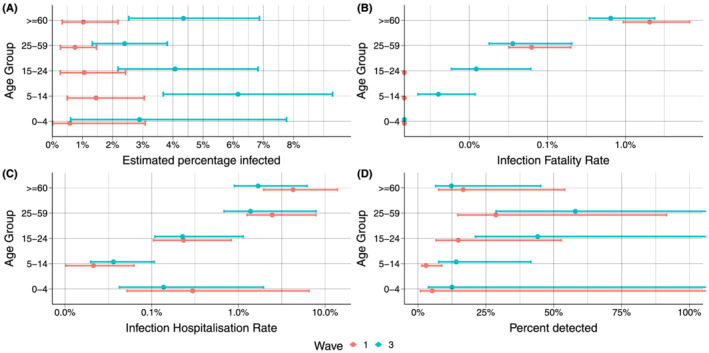
Estimated percentage infected, IFR, IHR and percentage detected infections by age and waves with 95% credible intervals. (A) Shows the estimated seroprevalence for the Norwegian population by age and round, (B–D) shows the infection fatality rate (IFR), infections hospitalization rate (IHR) and percentage of infections detected by age for Waves 1 and 2, respectively

**FIGURE 4:**
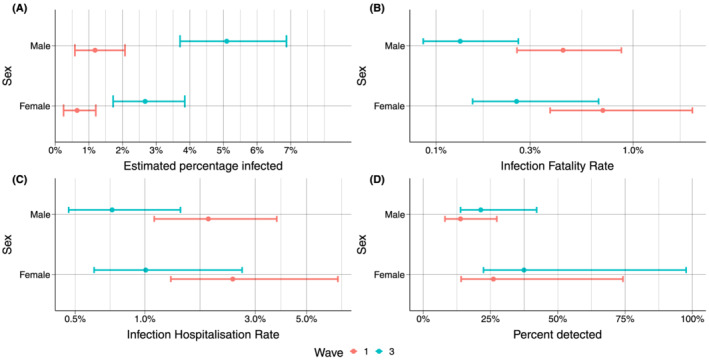
Estimated percentage infected, IFR, IHR and percentage detected infections by sex and waves with 95% credible intervals. (A) Shows the estimated seroprevalence by sex and collection round, (B–D) shows the infection fatality rate (IFR), infections hospitalization rate (IHR) and percentage of infections detected by sex for Waves 1 and 2, respectively

In Figures 3 and 4, we show the estimated percentage infected by wave and IFR, IHR, and percentage of infections detected by age and sex, respectively. Very few individuals below 15 years of age died or were admitted to hospital (Table 2). The IFR and IHR shows a clear increasing trend by age (Figure 3B,C, respectively), but the differences by wave are much smaller than for the overall IFR and IHR. The highest IFR and IHR was observed in individuals aged ≥60 years in the first wave (2.0% [95% CrI 1.0%–6.5%] and 4.1% [95% CrI 1.9%–12.9%], respectively). The percentage infections detected varied considerably between the age groups and was low for children and highest for the age group 25–59 years in both waves (28.4 and 57.7%, respectively) (Figure 3D).

The estimates of percentage infected for both the first and the second wave were higher for males, and the total numbers of COVID‐19‐associated deaths and hospitalizations, as well as confirmed cases, were also slightly higher in males than females (Table 2). However, due to the differences in estimated percentage of the population infected, females had a higher IFR, IHR, and percentage of detected infections for both waves (Figure 4).

## 4 DISCUSSION

We have conducted a repeated cross‐sectional study to monitor the seroprevalence over time in the Norwegian population during the first year of the COVID‐19 pandemic. The estimates were based on measurements of antibodies against SARS‐CoV‐2 in residual sera from microbiological laboratories. The estimated seroprevalence was found to be very low (less than 1%) immediately after the first peak of the pandemic in the spring of 2020 and in the late summer of 2020 (0.6%) (combined estimate of 0.8% after the first wave), followed by an increase in the estimated seroprevalence to 3.2% in January 2021, after the second wave.

Seroprevalence estimates of SARS‐CoV‐2 reported from Europe have varied considerably, both between and within countries, and depending on timing and the study populations.^15^ The seroprevalence development reported here for Norway was similar to what has been reported from Denmark, a neighbouring country with both a similar population size and comparable lock‐down measures (from 1.1% to 4.0% from May to December 2020).^16^

By mid‐May 2020, 8254 PCR‐confirmed cases of COVID‐19 (0.15% of the Norwegian population) had been notified,^17^ while the estimated seroprevalence indicated that 1% of the population had been infected.^10^ By the last week of July, the cumulative number of confirmed COVID‐19 cases had increased slightly to 9128 cases (0.17% of the population),^18^ while the seroprevalence estimate from the late summer was 0.6%. By late December, 47,462 cases of COVID‐19 (0.88% of the population) had been reported to MSIS,^19^ whereas the estimated seroprevalence indicates that 3.2% had been infected. Consequently, based on the estimated seroprevalence, the actual number of cases of SARS‐CoV‐2 infections in the Norwegian population may have been as much as three times higher than the confirmed number of cases.

In mid‐December 2020, the proportion of positive samples were 3.1% among a random sample of participants living in the area around Oslo, the capital of Norway.^20^ This is similar to our estimated national seroprevalence of 3.2%. A large seroprevalence study with 27 700 participants aged ≥16 years volunteering to be tested has been conducted in Norway.^21^ The seroprevalence was found to be 0.9% (95% CI 0.7%–1.0%) and the highest seroprevalence was found among teenagers aged 16–19 years. Although the samples of this study by Anda *et al*. and our study were collected in overlapping weeks, there was a 3‐fold difference in the proportion of positive samples reported. Differences may be partly explained by a healthy volunteer bias and not including children in the study by Anda *et al*., compared to an opposite bias towards residual sera being sampled from individuals with more morbid conditions in our study.^22^, ^23^ Thus, the true proportion of positives in Norway is likely to be somewhere between these estimates.

In Norway, few deaths were associated with COVID‐19 in 2020.^19^ By the end of 2020, the overall case fatality rate (CFR) was 0.9%. As can be expected, the IFR was lower than the reported CFR. Based on the estimated seroprevalence, the IFR was found to be 0.6% and 0.2% for the first and the second wave, respectively, for COVID‐19 infections in Norway. By 29 December 2020, the overall case hospitalization rate (CHR) due to COVID‐19 was 4.4%.^19^ We report an IHR of 2.3% in the first wave and 0.9% in the second wave. The estimated IFR for the first wave in Norway is quite similar to observations from other countries.^24^ Specifically, a Danish study reporting IFR and IHR by December 2020, reported an IFR of 0.53% and an IHR of 3.0%.^16^ We also observe a similar increase in IFR and IHR by age as reported by others.^16^, ^25^, ^26^

There was a large decrease in overall IFR during the second wave, but the decrease was much less evident by age‐group. The decrease in age‐specific IFR suggests that there were likely improvements in treatment, but the large change in the overall IFR is probably driven mainly by a change in the age distribution of infections between the two waves. When predominantly younger people are infected, as was the case for the second wave,^19^ overall IFR will typically be lower. This highlights the fact that comparisons of overall IFR can be hard to interpret. Due to non‐pharmaceutical interventions, the hospital capacity in Norway was never challenged, which might have contributed to the low IFRs, especially during the second wave. The IHR results follow a similar pattern with a large decrease in IHR from Waves 1 to 2 and a clear age trend. Others have reported that men have a higher IFR and IHR than women.^16^, ^27^ Our data suggest that women had slightly higher IFR and IHR; however, the total numbers of COVID‐19‐associated deaths and hospitalizations were low, and the differences were small.

In the beginning of the pandemic, only symptomatic individuals fulfilling certain criteria were tested, due to a shortage of test capacity and test reagents.^4^, ^19^ The test capacity improved during the summer of 2020, but it was still assumed that barely about 50% of cases would be detected through symptom‐based testing.^28^ We calculated the percentage of infections detected from the ratio of cases detected by PCR as reported to MSIS to our seroprevalence estimates. We find that the percentage of infections detected increased from 20% for Wave 1 to 28% for Wave 2, and this percentage varied considerably between age groups and males and females. The estimated detection percentages indicate that much fewer infections are detected among children through routine diagnostics than for adults, which also has been found by others.^29^ This could be related to a lower test frequency among children because children more often have asymptomatic and mild infections that may pass unrecognized and undiagnosed,^30^ as well as a possible hesitancy to let children undergo uncomfortable test procedures.

Seroprevalence studies may help to determine the number of SARS‐CoV‐2 infections in a population, as not all cases are tested and confirmed at the time of infection. However, there are several limitations to such studies.^31^ Individuals who are asymptomatic or have mild COVID‐19 may have lower levels of antibodies than individuals with severe disease.^6^, ^32^ If such low antibody levels are below the limit of detection for antibody assays, this may lead to underestimation of the seroprevalence. Furthermore, it is possible that not all individuals infected with SARS‐CoV‐2 will mount an antibody response. Antibodies against SARS‐CoV‐2 can be detected at least 8 months after infection, but the levels have been shown to decrease over time.^33^ Particularly, antibodies against the nucleocapsid protein wane faster than antibodies against the spike and RBD proteins.^34^ The seroprevalence estimates presented here did not consider waning of SARS‐CoV‐2 antibodies. In the present study, collection Rounds 1 and 2 occurred only a few months after the pandemic started. Moreover, most of the infections in Norway occurred in the autumn/early winter of 2020 just prior to collection Round 3. Waning was therefore not considered to play a significant role in this study but will, however, become increasingly important over time. Using residual sera to estimate population prevalence could lead to selection bias, as the samples came from medical laboratories, potentially including persons with more morbidity, comorbidities or different risk and health‐seeking behaviours compared with the general population. It should be noted that many of the participating laboratories receive patient samples for routine analysis of SARS‐CoV‐2 antibodies that may have been included in the sample selection. This could introduce a bias resulting in an increased positivity rate if samples received for this purpose are included. Conversely, testing of sera from invited persons may lead to volunteer healthy‐person biases or non‐participation of certain groups.^22^, ^23^ Thus, results from studies based on residual sera are not directly comparable with studies with different study designs. However, the consistent procedure of sampling of residual sera for the pre‐pandemic samples and the three sampling rounds makes the findings comparable over time.

## 5 CONCLUSION

In January 2021, the estimated seroprevalence for the Norwegian population was 3.2% (95% CrI 2.3%–4.1%), indicating that most of the population was still susceptible for SARS‐CoV‐2 infection after the first year of the COVID‐19 pandemic. The estimated seroprevalence suggests that the cumulative number of SARS‐CoV‐2 infections in Norway may have been approximately three times higher than the recorded number of confirmed cases by January 2021. The IFR and the IHR due to COVID‐19 were also low in the Norwegian population. Both rates increased with age and were lower in the second than in the first wave of the pandemic.

## AUTHOR CONTRIBUTIONS

**Gro Tunheim:** Conceptualization; project administration. **Gunnar Øyvind Isaksson Rø:** Conceptualization; data curation; formal analysis; methodology; validation; visualization. **Trung Tran:** Investigation. **Anne‐Marte Bakken Kran:** Conceptualization; project administration. **Jan‐Terje Andersen:** Methodology; supervision. **Eline Benno Vaage:** Investigation. **Anette Kolderup:** Resources. **John Torgils Vaage:** Methodology; resources; validation. **Fridtjof Lund‐Johansen:** Data curation; formal analysis; funding acquisition; investigation; methodology; resources; supervision; validation. **Olav Hungnes:** Conceptualization; data curation; investigation; project administration; resources; supervision.

## Data Availability

The data that support the findings of this study are available from the corresponding author upon reasonable request.
